# Assessment of Functioning of Health and Wellness Centers of Western Odisha: A Cross-Sectional Study

**DOI:** 10.7759/cureus.37665

**Published:** 2023-04-16

**Authors:** Smita K Panda, Devasish Panda, Ritesh R Behera, Sadhu C Panda, Aruna Munda, Pranab R Sahu

**Affiliations:** 1 Community Medicine, Veer Surendra Sai Institute of Medical Sciences and Research, Sambalpur, IND

**Keywords:** tele-consultation services, it infrastructure, rural phc hwcs, urban phc hwcs, comprehensive primary health care, health & wellness center

## Abstract

Background

The National Health Policy (NHP) 2017 of India recommends strengthening the delivery system of Primary Health Care through the establishment of Health & Wellness Centres (HWC) as the platform to deliver comprehensive primary health care services. HWCs are being set up as an upgraded version of existing sub-centers, primary health care centers, and urban primary health centers. This study was conducted to evaluate the functioning of health and wellness centers in Western Odisha.

Objective

To assess the availability of human resources, health care services, drug availability, lab services, and IT services at the health and wellness centers of Western Odisha.

Methods

Out of 10 districts of Western Odisha, two districts (Sambalpur and Deogarh) were selected for convenience, and a cross-sectional study was conducted from January 2021 to December 2022. All 43 health and wellness centers (35 rural primary health centers (PHCs) and eight urban PHCs) of the above two districts were included in the study. All relevant data were collected using a predesigned, pretested, and semi-structured questionnaire.

Results

The study showed that all 43 HWCs had good availability of pharmacists and lab technicians but less availability of medical officers, AYUSH medical officers, and staff nurses. Maternal and childhood services, family planning, and non-communicable disease (NCD) services were conducted regularly in all health and wellness centers, but basic oral health services and palliative care services were inadequate. All laboratory services like blood grouping, differential count/total leucocyte count, rapid test for pregnancy, urine albumin, urine routine examination/microscopic examination along with culture/sensitivity and water quality testing were done at urban PHC HWCs, whereas these lab services were less available at rural PHC HWCs. Drug groups like antipyretics, antihistaminic, antifungal, antihypertensive, oral hypoglycemic agent (OHA), antispasmodics, and antiseptic ointments were adequately available (>80%) at all urban and rural PHC HWCs. For IT support, appliances like desktops, internet facilities, and telephone facilities were found to be present at all HWCs. Teleconsultation services were found to be available at 88% of urban PHC HWCs and 60% of rural PHC HWCs.

​Conclusion

The study showed that infrastructure, human resources, and 12 service packages of health care and drugs should be addressed on a priority basis to achieve desired goals as envisaged by Ayushman Bharat to achieve the full potential of the health and wellness centers.

## Introduction

Health services are designed to meet the health needs of the community. There is global evidence that primary health care is critical to improving health outcomes. It has an important role in the primary and secondary prevention of several diseases, including non-communicable diseases. The provision of comprehensive primary health care reduces morbidity and mortality at much lower costs and significantly reduces the need for secondary and tertiary care. For primary health care to be comprehensive, it needs to span preventive, promotive, curative, rehabilitative, and palliative aspects of care. Primary health care goes beyond first contact care and is expected to mediate a two-way referral support to higher-level facilities (from primary to specialized care and back) and ensure follow up-support for individual and population health interventions [1].

But studies show that 11.5% of households in rural areas and about 4% in urban areas reported seeking any form of OPD care at or below the CHC level (except for childbirth) at primary care facilities, indicating low utilization of public health systems for other common ailments [2].

The National Health Policy (NHP) 2017 India recommends strengthening the delivery system of primary health care through the establishment of a health and wellness center (HWC) as the platform to deliver comprehensive primary health care services [3]. The first HWC was launched under the Ayushman Bharat program in Jangla village of Bijapur District of Chhattisgarh state in India on 14th April 2018 [4]. Till Date, 75,550 HWCs have been established, and the plan is to operationalize 1.5 lakh HWCs by 2022 [5].

HWCs are being set up as an upgraded version of existing subcenters, primary health care centers, and urban primary health care centers. Medical officers, along with MO AYUSH, pharmacist, laboratory technician, staff nurse, and ANM, are responsible for ensuring that CPHC services are delivered through all HWCs in their area. HWC is envisaged to deliver an expanded range of services that go beyond maternal and child health care services to include care for non-communicable diseases, palliative and rehabilitative care, oral, eye, ENT care, mental health, and the first level of care for emergencies and trauma, including free essential drugs and diagnostic services [6].

In the above context, the present study was undertaken to assess the availability of human resources, health care services, and drug availability at health and wellness centers of Sambalpur and Deogarh districts of Western Odisha.

## Materials and methods

Out of 10 districts in Western Odisha, two (Sambalpur and Deogarh) were selected for convenience. The study was conducted in 43 health and wellness centers (all rural PHCs and urban PHCs) in the Sambalpur and Deogarh districts of Western Odisha. Out of 43 health and wellness centers, 35 were from Sambalpur, and eight were from the Deogarh districts. A facility-based cross-sectional study was conducted for a period of two years, from January 2021 to December 2022. Ethical clearance was obtained from the Institutional Ethics Committee, i.e., VIREC (052-2022/I-S-T/26/Dt.17.05.2022). The permission letter was obtained from the CDM and PHO of the concerned districts before the initiation of the study. The process of collecting information was undertaken to find out the availability of human resources and health care services in all health and wellness centers in the aforementioned districts of Western Odisha. The data was collected using a predesigned and prestructured questionnaire according to the proforma for Ayushman-Bharat Health and Wellness Centre facility survey guidelines. The necessary information was collected from the available healthcare personnel posted at the health and wellness centers, viz., the medical officer, the AYUSH medical officer, the pharmacist, the staff nurse, the lab technician, the LHV, and the ANM. Data were entered in a Microsoft Office Excel v10 spreadsheet (Redmond, USA). Tables were prepared, and percentages were calculated for analysis.

## Results

Table 1 shows the comparison of the available human resources between rural and urban PHC HWCs. The human resources who were recommended to be present at HWCs were MO, MO AYUSH, pharmacist, lab technician, and ANM. Only 66% (170 out of 258) of the human resources were found in all HWCs of both districts. The total sanctioned strength of rural HWCs was 210, of which only 127 (60.4%) were found in position at the time of the survey. Similarly, out of 48 sanctioned posts, 43 (89.5%) urban HWCs had staff members in place. The strength of medical officers (63%) and AYUSH medical officers (77%), however, was found to be better in rural PHC HWCs than urban ones. Whereas paramedical staffs such as staff nurses, lab technicians, and ANMs were adequately present in the urban PHC HWCs. The posts of pharmacists were found completely filled up in both rural and urban PHC HWCs.

**Table 1:** Comparison of availability of human resources in rural vs. urban PHC-HWCs PHC: Primary Health Center, HWCs: Health and Wellness Centers, ANM: Auxiliary Nurse and Midwife

Sl No.	Human Resources	Rural (N=35)		Urban (N=8)		Total (N=43)	
		Sanctioned	Available N (%)	Sanctioned	Available N (%)	Sanctioned	Available N (%)
1	Medical Officer @ 1 per Facility	35	22 (63)	8	4 (50)	43	26(60)
2	MO AYUSH @ 1 per Facility	35	27 (77)	8	4 (50)	43	31(72)
3	Staff Nurse @ 1 per Facility	35	9 (29)	8	8 (100)	43	17(40)
4	Pharmacist @ 1 per Facility	35	35 (100)	8	8 (100)	43	43(100)
5	Lab Technician @ 1 per Facility	35	18 (51)	8	8 (100)	43	26(60)
6	ANM @ 1 per Facility	35	15 (43)	8	11 (>100)	43	27(53)
Total		210	127 (60.4)	48	43 (89.5)	258	170(66)

Table 2 explains the essential healthcare services (12 service packages ) that should be delivered at all HWCs. Out of the 12 service packages, three, like the management of communicable diseases according to the National Health Program’s diagnosis and management of NCDs, general OPD services, and minor ailments, were available at all 43 (100%) HWCs. Whereas emergency medical services, including burns and trauma care, were found to be available at only 12% of HWCs. It is also evident from Table 2 that most of the services were available at urban HWCs rather than rural ones. 

**Table 2:** Comparison of availability of health care services (12 service packages) in rural and urban PHC-HWCs RPHC: Rural Primary Health Center, UPHC: Urban Primary Health Center, NCD: Non-Communicable Disease

Sl. No	Services	RPHC (35) N (%)	UPHC(8) N (%)	Total (43) N (%)
1	Care in pregnancy and childbirth	16 (46)	4 (50)	20 (47)
2	Neonatal and infant health care services	16 (46)	4 (50)	20 (47)
3	Childhood and adolescent health care services	28 (80)	8 (100)	36 (84)
4	Family planning, contraceptive services, and other reproductive health care services	34 (97)	8 (100)	42 (98)
5	Management of communicable disease: National Health Programs	35 (100)	8 (100)	43 (100)
6	General out-patient care for acute simple illness and minor ailments	35 (100)	8 (100)	43 (100)
7	Screening, prevention, control, and management of NCD	35 (100)	8 (100)	43 (100)
8	Care for ophthalmic and ENT problems	0	8 (23)	8 (19)
9	Basic oral health care services	8 (23)	5 (63)	13 (30)
10	Elderly and palliative health care services	13 (37)	5 (63)	18 (67)
11	Emergency medical services, including burns and trauma	0	5 (63)	5 (12)
12	Screening and basic management of mental health ailments	3 (9)	7 (88)	10 (23)

Table 3 compares the availability of different laboratory services between rural and urban PHC HWCs of Sambalpur and Deogarh districts. Laboratory services like blood grouping, DC/TLC, rapid tests for pregnancy, urine albumin, urine RE/ME and C/S, and water quality testing were done at all urban PHC HWCs. Tests for malaria (R-D kit) were available at all 43 HWCs (both rural and urban). Gram staining for RTI/STD (9%) and water quality testing (11%) were found least available. Lab services at HWCs are present in rural areas, whereas platelet count (50%) is the least available service in urban HWCs.

**Table 3:** Comparison of availabilities of different laboratory services in rural and urban PHC-HWCs DC: Differential Count, TLC : Total Leukocyte Count,  STD: Sexually Transmitted Disease, RE/ME : Routine Examination/ Microscopic Examination, C/S : Culture/ Sensitivity

Sl. No	Laboratory Services	RPHC (35) N (%)	UPHC (8) N (%)	Total (43) N (%)
1	Blood Group	11 (31)	8 (100)	19 (44)
2	DC/TLC	16 (46)	8 (100)	24 (56)
3	Platelet Count	5 (14)	4 (50)	9 (21)
4	Gram Staining for diagnosis of RTI/STD	3 (9)	5 (63)	8 (19)
5	Malaria RD Kit	35 (100)	8 (100)	43 (100)
6	Urine Albumin	10 (29)	8 (100)	18 (42)
7	Urine RE/ME & C/S	9 (31)	8 (100)	17 (40)
8	Rapid Test for Pregnancy	26 (74)	8 (100)	34 (49)
9	Water quality Testing	4 (11)	8 (100)	12 (28)

Table 4 compares IT infrastructure and teleconsultation services between rural and urban HWCs. IT support is mainly necessary for telemedicine services, i.e., E-Sanjeevani. E-Sajeevani is a low-cost, integrated telemedicine application developed by Ayushman Bharat. This web-based application provides consultation with PHCs and higher referral centers as a hub-and-spoke model. Out of the 43 HWCs in both the above districts, the availability of desktops and tablets was 95% and 5%, respectively. Internet facilities were found in all urban HWCs, but they were found only in 69% of rural HWCs. Teleconsultation services were found to have been started in 88% of urban PHC HWCs, whereas they were found in only 60% of rural PHC HWCs, mainly due to logistical and infrastructural constraints.

**Table 4:** Comparison of availability of IT infrastructure and teleconsultation services in rural and urban PHC-HWCs IT: Information Technology, CUG: Closed User Group

Sl.no	IT Infrastructure	RPHC HWC (35) N (%)	UPHC (8) N (%)	Total (43) N (%)
1	Desktop/Laptop	33 (94)	8 (100)	41 (95)
2	Internet Facility	24 (69)	8 (100)	32 (74)
3	Telephone (CUG) Facillity	17 (49)	5 (63)	22 (52)
4	Tablet	1 (3)	1 (13)	2 (5)
5	Teleconsultation Services	21 (60)	7 (88)	28 (65)

Table 5 shows the information on the availability of drug groups at HWCs (rural and urban PHCs) in Sambalpur and Deogarh, according to the essential drug list. During the survey, the presence of at least 50% of drugs from the particular group is considered "adequate". All rural PHC HWCs of Deogarh and urban PHC HWCs of Sambalpur district were found to have at least 50% of the advised drugs on the list at the time of the survey. Except for the PPI and analgesic groups, all 43 HWCs in both districts were found to have at least 50% of the advised drugs from their respective drug groups. 

**Table 5:** Comparison of availability of drugs from essential drug list in rural and urban PHC-HWCs OHA: Oral Hypoglycemic Agent, PPI: Proton Pump Inhibitor

Sl.no	Drug Group	RPHC (35) N (%)	UPHC (8) N (%)	Total (43) N (%)
1	Antipyretic	35 (100)	8 (100)	43 (100)
2	Analgesic	24 (69)	8 (100)	32 (74)
3	Antibiotic	27 (77)	8 (100)	35 (81)
4	Antihistaminic	32 (91)	8 (100)	40 (93)
5	Antifungal	34 (97)	8 (100)	42 (98)
6	Antihypertensive	33 (94)	8 (100)	41 (95)
7	OHA	31 (89)	8 (100)	39 (91)
8	Antiemetic	32 (91)	8 (100)	40 (93)
9	PPI	22 (63)	8 (100)	30 (70)
10	Antispasmodic	32 (91)	8 (100)	40 (93)
11	Antiseptic Ointment	35 (100)	8 (100)	43 (100)

## Discussion

As per the IPHS guideline, a PHC should have one medical officer, one AYUSH medical officer, one staff nurse, one pharmacist, one lab technician, and one ANM. In our study, 170 (66 %) (Table 1) staff was working against the sanctioned posts. In our study, 67% of staff were working, which was also true in the study “Assessment of Health & Wellness Centers conducted by the State Health Resource Centre Madhya Pradesh [7]. 

In this study, services like emergency medical services, including burns and trauma, were found to be most affected by the unavailability of trained human resources, followed by care for ophthalmic and ENT problems, screening and basic management of mental health ailments, and basic oral health care. At the same time, services like family planning, contraceptive services, and other reproductive health care services, management of communicable diseases through the National Health Programs, general out-patient care for acute simple illnesses and minor ailments, screening, prevention, control, and management of NCDs were available at all HWCs. But care in pregnancy and childbirth, as well as neonatal and infant health care services were available at 47% of HWCs (Table 2). However, neonatal and infant health care services were available at 91% of HWCs, while child and adolescent health services, along with family planning and contraceptive services, were available at 93% of each HWC, respectively, in the study "Assessment of Health & Wellness centers in Madhya Pradesh" [7].

Teleconsultation services are very essential for strengthening healthcare delivery. These teleconsultation services require logistics like desktops, tablets, and internet facilities. Therefore, information on the availability of the MO portal for teleconsultation services was obtained. Desktop facilities were found to be available at 95% of HWCs in both districts, whereas tablet availability was found at 5% in both districts. The percentage of HWCs with internet access was 74% in both districts (Table 4). Similarly, desktop facilities were available in 89% of HWCs and internet facilities in 100% of HWCs in the study by the Assessment of Health and Wellness Centers in Madhya Pradesh [7].

The availability of drugs at HWCs is essential for patients. In our study, the availability of more than 50% of drugs from each group was considered adequate. Most of the drugs from each group were found to be present at the time of the survey. Analgesics and PPIs were the least available drugs at HWCs due to the high prescription rate (fast-moving). Antihypertensive drugs like Amlodipine and Telmisartan were also present at HWCs in both districts. Antihypertensives like Amlodipine and Telmisartan were present in 93% of rural HWCs and 100% of urban HWCs in Sambalpur and Deogarh districts, respectively. The oral hypoglycaemic agent (OHA) group of drugs like tab Metformin and tab Glimiperide were present at HWCs in both Sambalpur and Deogarh. The OHA group of drugs was present in 23 (85%) rural HWCs and 43 (100%) urban HWCs (Table 5).

## Conclusions

In our study, a shortage of healthcare staff was the key drawback in service delivery. During the survey, only 66% of human resources were functioning. Out of 12 service packages of health services, family planning, and other contraceptive services, management of communicable disease through the National Health Program, general outpatient care for simple illnesses, screening, prevention, control, and management of NCDs were most often available at all facilities. Basic oral health care services and elderly palliative and rehabilitative care services were not fully functional at many HWCs in both districts. For IT support and teleconsultation services, internet facilities were available at most of the HWC facilities, but the availability of tablets (ANMOL)/mobile phones for the CHO, ANM, and medical officer was not adequate, which hampers the digitalization of data. Medicines were most often available at all facilities, but at a few facilities, basic medicines like analgesics and PPIs were not present in sufficient amounts. Space for recreational activities like yoga was not present in many facilities. So, the infrastructure of HWCs should be modified as per IPHS 2022, and staff should be posted as per the IPHS norm for the provision of quality comprehensive services. It is, therefore, recommended that the identified gaps, including infrastructure, human resources, and 12 service packages of health care services and drugs, be addressed on a priority basis to achieve the desired goals as envisaged by Ayushman Bharat and achieve the full potential of the health and wellness centers. 
